# Stable Immune Response Induced by Intradermal DNA Vaccination by a Novel Needleless Pyro-Drive Jet Injector

**DOI:** 10.1208/s12249-019-1564-z

**Published:** 2019-12-09

**Authors:** Chinyang Chang, Jiao Sun, Hiroki Hayashi, Ayano Suzuki, Yuko Sakaguchi, Hiroshi Miyazaki, Tomoyuki Nishikawa, Hironori Nakagami, Kunihiko Yamashita, Yasufumi Kaneda

**Affiliations:** 1 Department of Device Application for Molecular Therapeutics, Osaka University Graduate School of Medicine, Suita-shi, Osaka, Japan; 2 Department of Health Development and Medicine, Osaka University Graduate School of Medicine, Suita-shi, Osaka Japan; 3 Medical Device Division, Daicel Corporation, R&D Headquarters, Minato-ku, Tokyo, Japan

**Keywords:** DNA vaccine, vaccine delivery, animal experiments, pyro-drive, jet injector

## Abstract

DNA vaccination can be applied to the treatment of various infectious diseases and cancers; however, technical difficulties have hindered the development of an effective delivery method. The efficacy of a DNA vaccine depends on optimal antigen expression by the injected plasmid DNA. The pyro-drive jet injector (PJI) is a novel system that allows for adjustment of injection depth and may, thus, provide a targeted delivery approach for various therapeutic or preventative compounds. Herein, we investigated its potential for use in delivering DNA vaccines. This study evaluated the optimal ignition powder dosage, as well as its delivery effectiveness in both rat and mouse models, while comparing the results of the PJI with that of a needle syringe delivery system. We found that the PJI effectively delivered plasmid DNA to intradermal regions in both rats and mice. Further, it efficiently transfected plasmid DNA directly into the nuclei, resulting in higher protein expression than that achieved *via* needle syringe injection. Moreover, results from animal ovalbumin (OVA) antigen induction models revealed that animals receiving OVA expression plasmids (pOVA) *via* PJI exhibited dose-dependent (10 μg, 60 μg, and 120 μg) production of anti-OVA antibodies; while only low titers (< 1/100) of OVA antibodies were detected when 120 μg of pOVA was injected *via* needle syringe. Thus, PJI is an effective, novel method for delivery of plasmid DNA into epidermal and dermal cells suggesting its promise as a tool for DNA vaccination.

**Electronic supplementary material:**

The online version of this article (10.1208/s12249-019-1564-z) contains supplementary material, which is available to authorized users.

## INTRODUCTION

Technology is constantly changing and advancing with many novel devices being discovered with potential to serve as preventative or therapeutic strategies for various diseases. For more than two centuries, vaccination has been widely employed as an effective medical technology. However, although many types of vaccines have proven effective, a need remains for the modification and creation of simpler and more effective injection technologies [1].

Many factors affect the efficacy of vaccines, including delivery route and the resulting immune response. Injection zones differ depending on vaccine function, with many injected either intramuscularly or subcutaneously. Additionally, considering the initial immune stimulation induced by vaccines, the distribution of antigen-presenting cells (APCs) is considered an important element for vaccine effectiveness. Since APCs function to process the antigen, present epitopes on the cell surface, and stimulate other immune cells, including T lymphocytes, their induction is considered a primary and essential step for effective immunization. As such, the intradermal region is considered to be an optimal target for vaccine delivery due to ease of access for APCs such as Langerhans’s cells (LH cells) and dermal dendritic cells within this region. However, intradermal injection is technically difficult for non-professionals to perform; hence, to overcome this obstacle, needleless injection systems, such as the micro-needle or jet injector, have been developed and employed [2–4].

Over the past decade, DNA-based vaccines (DNA vaccines) have been developed offering several advantages to traditional protein or peptide-based vaccines. First, production of DNA vaccines does not require cultivation of the target pathogen; and second, they can be produced on an industrial scale [5, 6]. However, inherent obstacles are associated with delivery of DNA vaccines. Compared to traditional protein vaccines, DNA vaccines require not only accurate physical injection, but also efficient gene expression following injection. To overcome these challenges, two primary types of *in vivo* DNA transfer devices have been designed. The first is an electroporation (EP) system, which requires needle syringe injection of DNA into the target region followed by EP of the DNA into cells [7]. The second strategy involves a mechanically powered jet injection system that performs DNA delivery to the target tissue in one step. Currently, powered jet-injection systems are most commonly used for intramuscular or subcutaneous administration; however, they have also been developed for intradermal injection [1, 8–11]. Alternatively, combinatorial strategies involving jet injection followed by EP have been examined for use in efficient DNA vaccine delivery [12–14]. More recently, a sophisticated new type of needleless device was reported to control the injection depth and speed using a computer-controlled motor system and an electrical feedback system; however, there has yet to be reports on its application for DNA vaccination [15]. Thus, the development of novel DNA vaccines requires testing of new devices specific for intradermal DNA vaccination on experimental animal models. Many previous reports on jet injectors proposed for intramuscular and subcutaneous administration suggest possible injection volumes of 0.1 to 1 mL. However, these volumes are considered too high for accurate intradermal injection, as the skin thickness was reported to be only 0.2 mm for mice and 2 mm for rats. Alternatively, the potential injection volumes of the new device examined within this study were determined to be 0.01 to 0.1 mL.

Although existing delivery devices have been shown to effectively transport DNA intra-muscularly resulting in adequate expression, the development of an efficient DNA delivery system for intradermal vaccination has yet to be described. In this study, we developed and tested a pyro-drive jet injector (PJI) for DNA vaccination with a particular focus on its ability to effectively adjust injection depth, deliver DNA directly into the intradermal region of experimental rats and mice, induce gene expression, and for the production of stable antibodies.

## MATERIALS AND METHODS

### Animals

Female 6–10-week-old CD (Sprague Dawley; SD) rats (Charles River Japan Inc., Kanagawa, Japan) and BALB/c mice (CLEA Japan Inc., Tokyo, Japan) were used in the study. All animals were maintained under controlled conditions (temperature, 21.0–24.5°C; humidity, 45 ± 15%; ventilation, 8–15 times/h; light/dark cycle, 12 h) in a pathogen-free room. Animals received *ad libitum* food and water and were handled according to the approved protocols of the Animal Committee of Osaka University (Suita, Japan) and the Ethics Committee for animal experiments of the Safety Research Institute for Chemical Compounds Co. Ltd. (Sapporo, Japan).

### Optimization of Intradermal Injection Conditions

To determine the suitable ignition powder mass for rats, the animals were anesthetized. India ink (Kaimei & Co., Ltd. Saitama, Japan) was diluted twice with distilled water before injecting 30 μL *via* PJI (DAICEL Corporation, Osaka, Japan) into the right flank using various doses of ignition powder (15, 35, 55, 75, or 90 mg) with 40 mg smokeless powder. The mice were also injected with 10 μL of diluted India ink (diluted 10 times with distilled water before injection) into the right flank area using 15, 25, 35, or 45 mg of ignition powder with 40 mg smokeless powder. The ignition powder mass affects the distribution depth and smokeless powder affects the injection volume of India ink [16]. After injection, the tissues were collected and fixed with 10% formalin (Nacalai Tesque, Inc., Tokyo, Japan) for histopathological analysis. The target injection area is illustrated in Supplementary Fig. 1.

### Intra-Nuclear Plasmid DNA Delivery by Pyro-Drive Jet Injector

Cy3-labeled plasmid DNA (Cy3-p) at 0.5 μg/μL (Takara Bio Inc., Shiga, Japan) was injected into the right flank area with the PJI or needle syringe using anesthetized rats (30 μL) and mice (10 μL). A combination of 35 mg ignition powder and 40 mg smokeless powder was used for rats, whereas a combination of 25 mg ignition powder and 40 mg smokeless powder was used for mice. Needle syringes of 27G and 30G were used for rats and mice, respectively. The diameter of the PJI injection nozzle was roughly equivalent to 30G for both rats and mice. After injection, the tissue was excised from an approximate 1-cm^2^ region and immediately frozen in OCT compound (Sakura Finetek Japan Co., Ltd., Tokyo, Japan) and sectioned at − 20°C, which is the optimal cutting temperature. To analyze plasmid DNA delivery, sectioned skin samples were stained with ProLong Gold Antifade Mountant with DAPI (Thermo Fisher Scientific, MA, USA) and observed using a fluorescence microscope (BZ-X700; Keyence, Osaka, Japan). When the Cy3 and DAPI fluorescence signal overlap was > 50%, Cy3 was considered to be introduced into the nuclear directory after injection.

### Evaluation of Skin Injury after Plasmid DNA Injection

To assess the influence of plasmid DNA injection in the intradermal region, pGL3 firefly luciferase expression plasmid (Luc plasmid) DNA (1 μg/μL) (Promega Corporation, WI, USA) was dorsally injected (30 μg) using the PJI and needle syringe (27G) into the right flank area of rats, and dorsally injected (10 μg) using the PJI and needle syringe (30G) in mice. Combinations of 35 mg ignition powder and 40 mg smokeless powder for rats, and 25 mg ignition powder and 40 mg smokeless powder for mice were used. After injection, skin samples were collected at 0, 6, and 24 h. The excised skin sections were fixed with 10% formalin and embedded in paraffin. Histological examinations were performed based on hematoxylin–eosin (HE) staining. HE staining procedures were subsequently performed on deparaffinized sections.

### Gene Expression and Kinetic Analysis of Injected Plasmid DNA

#### Time Course of Gene Expression

To compare gene expression between PJI and needle syringe methods, the Luc plasmid (1 μg/μL) was dorsally injected into rats (*n* = 4; 30 μg/animal) and mice (*n* = 6; 10 μg/animal) on the right and left flank areas using the PJI and needle syringes. Combinations of 35 mg ignition powder and 40 mg smokeless powder for rats, and 25 mg ignition powder and 40 mg smokeless powder for mice were used. After injection, the plasmid DNA-injected skin regions were punched out with a 5-mm (in diameter) biopsy punch (Kai Industries Co Ltd., Seki, Japan) every 24 h for 72 h. After skin sample collection, a luciferase assay was detected using a Luciferase assay kit (Promega) according to the manufacturer’s instructions. Relative light units (RLU) were measured using a lumitester C-110 (Kikkoman Biochemifa Company, Tokyo, Japan).

#### Quantitative Analysis of Injected Plasmid DNA

The injection conditions for the Luc plasmid (30 μg) in SD rats (*n* = 3) were the same as those for the gene expression experiments described previously herein. After the tissue samples were collected, total DNA was purified using a NucleoSpin DNA purification kit (Macherey-Nagel, Germany) according to the manufacturer’s instructions. Luciferase gene copy number was determined using a luciferase gene specific Taq-man probe (ID Mr0398758_mr) and an Applied Biosystems 7900HT PCR System (Thermo Fisher Scientific).

### Model DNA Vaccination Experiment

#### Ovalbumin (OVA) Gene Expression

Dosages of either 3 μg (0.1 μg/μL), 10 μg (0.33 μg/μL), or 30 μg (1 μg/μL) pOVA (pcDNA3-OVA; pcDNA3-OVA was a gift from Sandra Diebold & Martin Zenke; Addgene plasmid no. 64599, http://n2t.net/addgene:64599, RRID = Addgene_64,599; Addgene, MA, USA) were injected into the right flank area of SD rats using the PJI and needle syringe (*n* = 3 for each device) [17]. Twenty-four hours after injection, the plasmid DNA-injected skin region was punched out with a 5-mm (in diameter) biopsy punch and total protein was extracted using the same methods as in the luciferase assay protocol. OVA and total protein were quantified using an OVA ELISA Kit (ITEA Inc., Tokyo, Japan) and Bio-Rad protein assay dye reagent concentrate (Bio-Rad Laboratories, CA, USA), respectively, according to the manufacturers’ instructions.

#### OVA-Specific Antibody Production by pOVA Injection

Dosages of 10 μg pOVA (0.3 μg/μL, 30 μL/injection), 60 μg pOVA (1 μg/μL, 30 μL × 2 injections/rat), and 120 μg pOVA (1 μg/μL, 30 μL × 4 injections/rat) were injected into the right flank area of SD rats (*n* = 4) a total of three times over a 2-week period (at weeks 0, 2, and 4) using the PJI, and 120 μg pOVA (1 μg/μL, 30 μL × 4 injections/rat) was injected into the right flank area of SD rats (*n* = 4) a total of three times over a 2-week period (at weeks 0, 2, and 4) using a needle syringe (27G). Serum was collected before injection, and then again every 2 weeks from 0 to 8 weeks.

To compare antibody production, levels were compared after pOVA injection by the PJI and those induced by OVA protein delivered *via* the needle syringe (27G). For this, 10 μg pOVA, (0.3 μg/μL, 30 μL/injection) and 120 μg pOVA (1 μg/μL, 30 μL × 4 injections/rat) were injected by the PJI, and 60 μg OVA (1 μg/μL, 30 μL × 2 injections/rat) was injected using the needle syringe to SD rats (*n* = 4 for 60 μg OVA group and *n* = 5 for other groups) a total of three times over a 2-week period. Serum was collected before injection, and then again every 2 weeks from 0 to 8 weeks. To assess antibody production stability, 60 μg of pOVA (1 μg/μL, 30 μL × 2 injections/rat) was injected by the PJI using two different quantities of ignition powder, 35 or 90 mg (*n* = 3), into the right flank area a total of three times over a 2-week period (0, 2, and 4 weeks), and serum was collected before every injection until 6 weeks (0, 2, 4, and 6 weeks). A total of 60 μL of 10 mM Tris–1 mM EDTA (TE) solution (Qiagen N.V., PL, NLD) was injected by the PJI as a negative control for all experiments. In the mouse model, 10 μg (0.5 g/μL), 3.3 μg (0.17 g/μL), and 1 μg (0.05 g/μL) pOVA were injected into the right flank area of Balb/c mice (*n* = 4) a total of three times over a 2-week period (at weeks 0, 2, and 4) using the PJI and a needle syringe (30G). Serum was collected before injection, and then every 2 weeks from 0 to 8 weeks. A TE solution was injected with the PJI as a negative control. Anti-OVA specific antibody levels were analyzed by ELISA.

#### OVA Antibody ELISA

Serum was collected from rats on days 0, 14, and 28 before pOVA injection and on day 42 for all vaccination studies, as well as on day 56 for the antibody production stability study. The collected serum samples were stored at − 80°C until analysis. The recombinant OVA protein (Wako Pure Chemicals Industries Ltd., Tokyo, Japan) was coated onto ELISA plates at 10 μg/mL in a carbonate buffer incubated overnight at 4°C. Serum samples were diluted from 10- to 31,250-fold in PBS containing 5% skim milk and then incubated on plates at 4°C overnight. After serum culture, HRP anti-rat IgG (GE Healthcare Life Sciences, PA, USA) was incubated with the samples for 3 h at room temperature (24°C), and color development was performed with the peroxidase chromogenic substrate 3,3′-5,5′-tetramethyl benzidine (Sigma-Aldrich Co., St Louis, MI, USA). Absorbance was detected using a microplate reader (Bio-Rad Laboratories, Inc., Hercules, CA, USA) at 450 nm. OVA antibody ELISA for mouse serum was performed according to the rat serum ELISA procedure save for the detection antibody. HRP anti-mouse IgG (Promega Corporation, WI, USA) was employed for the mouse model.

#### Titer Calculation

The cut-off value was calculated using the OD_450_ value from ELISA using the serum obtained from the TE-injected group. The average OD_450_ value was applied as follows: cut-off value = OD_450_ average × 2 and the antibody titer was equal to the dilution fold at the cut-off point, calculated as the antibody titer of serum samples using two-point calibration.

### Statistical Analysis

A non-parametric Shirley–Williams test and one-way analysis of variance, followed by Dunnett’s test were used to evaluate statistical significance using BellCurve for Excel (Social Survey Research Information Ltd., Tokyo, Japan). *P* values less than 0.05 were considered statistically significant (*p < 0.05, **p < 0.01).

## RESULTS

### Optimization of Intradermal Injection Conditions

**Fig. 1:**
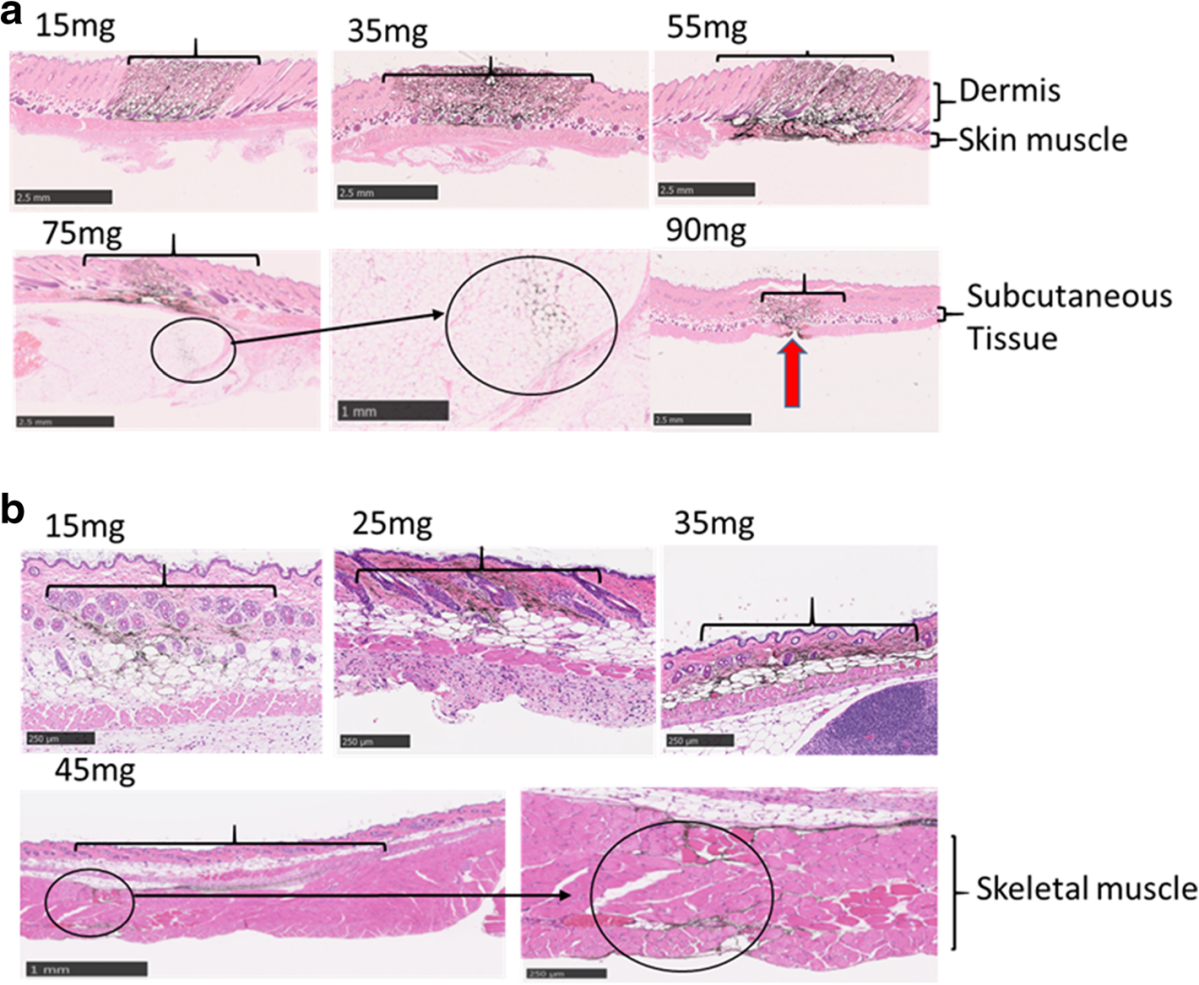
Distribution of pyro-drive jet injector (PJI)-injected India ink under different ignition powder conditions in rat and mouse models. India ink (black) was injected using different amounts of PJI ignition powder into rats (**a**) and mice (**b**). (**a**) In the rat model, conditions using 15, 35, 55, 75, and 90 mg of PJI ignition powder were assessed. (**b**) In the mouse model, 15, 25, 35, and 45 mg of PJI ignition powder were assessed. Skin samples were collected immediately and treated with H&E staining. Red arrow: observed break point in skin muscle. Scale bars in (**a**) were 2.5 mm except for the close-up figure (1 mm). Scale bars in (**b**) were 250 μm save for the left panel showing 45 mg, which was 1 mm. Brackets indicate the width of diffusion. Red arrows indicated the breaking point of skin muscle

The sample injection depth is adjustable based on the amount of ignition powder [16]. Hence, we initially screened suitable ignition powder masses (15–90 mg) in both rats and mice. The histopathological results are depicted in Fig. 1a. India ink primarily diffused into the rat dermal region following use of 15 and 35 mg ignition powder (Fig. 1a). However, when 15 mg ignition powder was applied, a greater amount of India ink remained on the skin surface compared to when 35 mg of ignition powder was used (visual observation, data not shown). In addition, when 55, 35, and 15 mg ignition powder were used for rat injection, India ink was observed in the intradermal region and subcutaneous tissue; however, the distribution patterns differed. With 55 mg, the India ink diffused into the intradermis, subcutaneous tissue, and skin muscle regions. When high ignition powder quantities (75 and 90 mg) were used, the injected India ink reached the rat musculi trunci region, permeating the skin and subcutaneous tissue including the lipocyte tissue region and skin–muscle layers. In mice (Fig. 1b), ignition powder quantities from 15 to 45 mg were used. When 25 mg ignition powder was used, the ink was observed within the dermis and subcutaneous tissue. With ignition powder amounts greater than 25 mg (35 and 45 mg), the India ink was present throughout the dermis and subcutaneous tissue, including the lipocyte tissue and dermal-muscle layers, and reaching the musculi trunci region. Thus, the optimum conditions for intradermal injection were 35 mg for rats and 25 mg for mice.

### Dermal Plasmid DNA Delivery and Intra-Nuclear Plasmid DNA Delivery by PJI

**Fig. 2:**
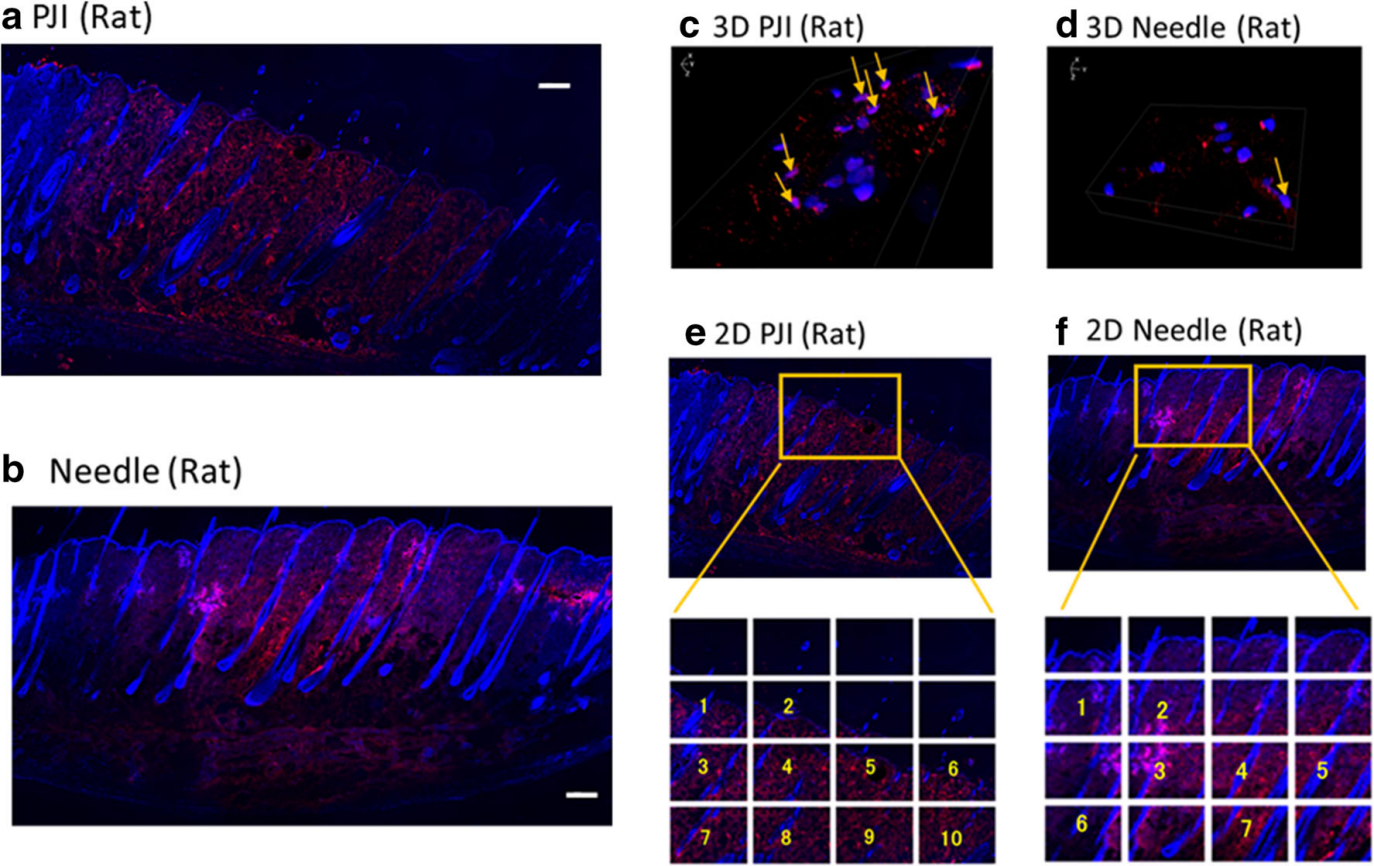
Plasmid distribution following pyro-drive jet injector (PJI) and needle syringe injection. Cy3-labeled plasmid DNA (30 μL, 0.5 mg/mL) was injected *via* PJI (35 mg ignition powder) (**a**) and needle syringe (27G needle) (**b**) into a rat model. Plasmid DNA distribution 3D analysis (**c**, **d**) and 2D analysis (**e**, **f**) are shown. Yellow arrows: nuclei and plasmid DNA overlapping sites. Yellow box: nuclei and plasmid DNA overlap analysis by region (detailed in Table 1). Cy3: red; DAPI: blue. Scale bars (white) in (**a**) and (**b**) indicate 300 μm. Needle: needle syringe

Suitable injection conditions were identified for rats and mice (Fig. 1); the optimal ignition powder mass for intradermal injection in rats and mice was 35 and 25 mg, respectively. By comparing plasmid DNA delivery between needle syringe and PJI, we found that the PJI group exhibited evenly distributed Cy3-p in the epidermal and dermal regions, whereas needle syringe injection primarily resulted in plasmid DNA distribution in the dermal to subcutaneous regions (Fig. 2a, b). Furthermore, the PJI group showed a large degree of Cy3-p overlap with the nucleus as demonstrated by 3D imaging in rat skin samples (Fig. 2c, d). In the PJI group, the level of Cy3-p transported into the nuclei immediately after injection was high (average 64.9%, peak 97.1%); however, it was low in the needle syringe group (average 10.4%, peak 25%) resulting in a statistically significant difference between these two delivery groups (*p* < 0.01) (Fig. 2e, f and Table 1). Similarly, within the mouse study, the average and peak values were 23.5% and 44.7% for PJI and 3.5% and 7.7% for needle syringe, respectively. These results suggested that PJI effectively introduced plasmid DNA into the nucleus at a high frequency in rat and mouse dermal tissue (mouse data not shown).

**Table 1:** Cy3-p Introduction Ratio of PJI and Needle Syringe

	Region	1	2	3	4	5	6	7	8	9	10	Avg.
%	PJI	60.0	75.0	54.9	57.9	74.4	97.1	52.1	54.1	58.8	65.1	64.9
	Needle	6.8	3.2	8.3	6.3	8.7	25	14.3	N.D.	N.D.	N.D.	10.4

Ratio of detected overlap of nuclei (DAPI) and injected Cy3 plasmid DNA after pyro-drive jet injector (PJI) and needle syringe injection shown in Fig. 2e and f

% = Cy3 + DAPI/DAPI, Avg. = average nuclear Cy3 induction, N.D. = not detected

### Evaluation of Skin Injury after Plasmid DNA Injection

**Fig. 3:**
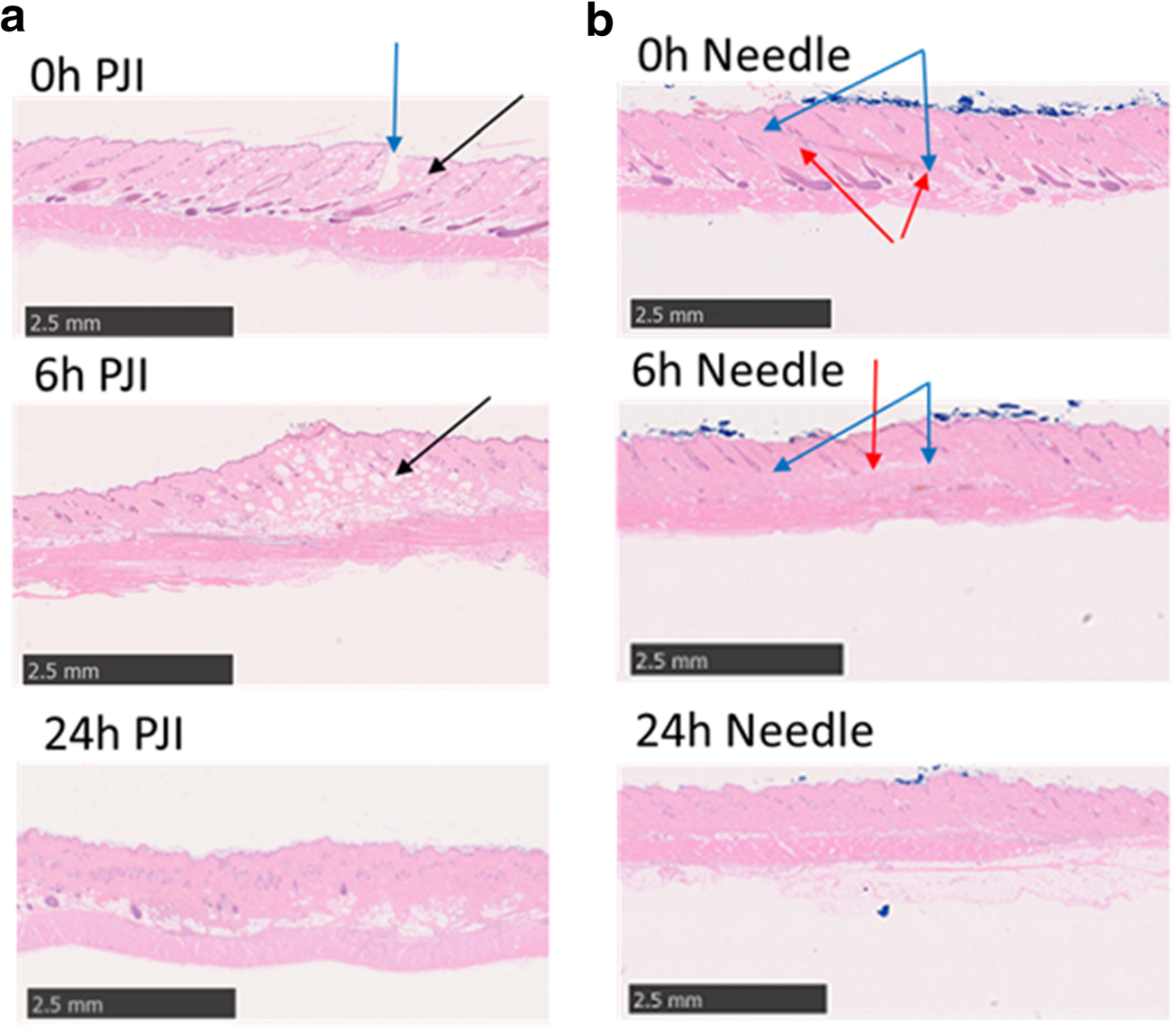
Time course of skin damage test. Luciferase plasmid DNA was injected using 35 mg of ignition powder by a pyro-drive jet injector (PJI) (**a**) or 27G needle syringe (**b**). After injection, the skin samples were collected at 0 h (just after injection), 6 h, and 24 h and stained using H&E. Blue arrows: visible puncture point. Black arrows: spherical cleavages. Red arrows: bleeding site

For an accurate evaluation of the delivery system, not only delivery efficacy but also damage at the injection site are important considerations. To evaluate the utility of PJI as a DNA vaccination tool, we evaluated skin damage after PJI injection with a Luc plasmid. Figure 3 shows the degrees of skin damage after PJI or needle syringe injection in a rat model in terms of histopathological analysis results at 0, 6, and 24 h after Luc plasmid DNA injection. PJI delivery caused small spherical cleavages and swelling that were observable until 6 h (Fig. 3a). However, the small spherical cleavages and swelling disappeared by 24 h; no other remarkable intradermal inflammation or bleeding were observed at any observation point. Alternatively, needle syringe delivery resulted in intradermal bleeding from the puncture wound observed at 6 h (Fig. 3b). However, no remarkable adverse events were observed at 24 h for either injection method. These results show that PJI did not induce skin damage complications after plasmid DNA injection and did not induce bleeding at the injection site, as compared to the needle syringe. In mice, similar results were observed as for the mice; specifically, skin damage induced by the PJI was not excessive compared to that observed with needle injection (Supplementary Fig. 2).

### Luciferase Gene Expression and Copy Number Evaluation of Injected Plasmid DNA in the Skin Region

**Fig. 4:**
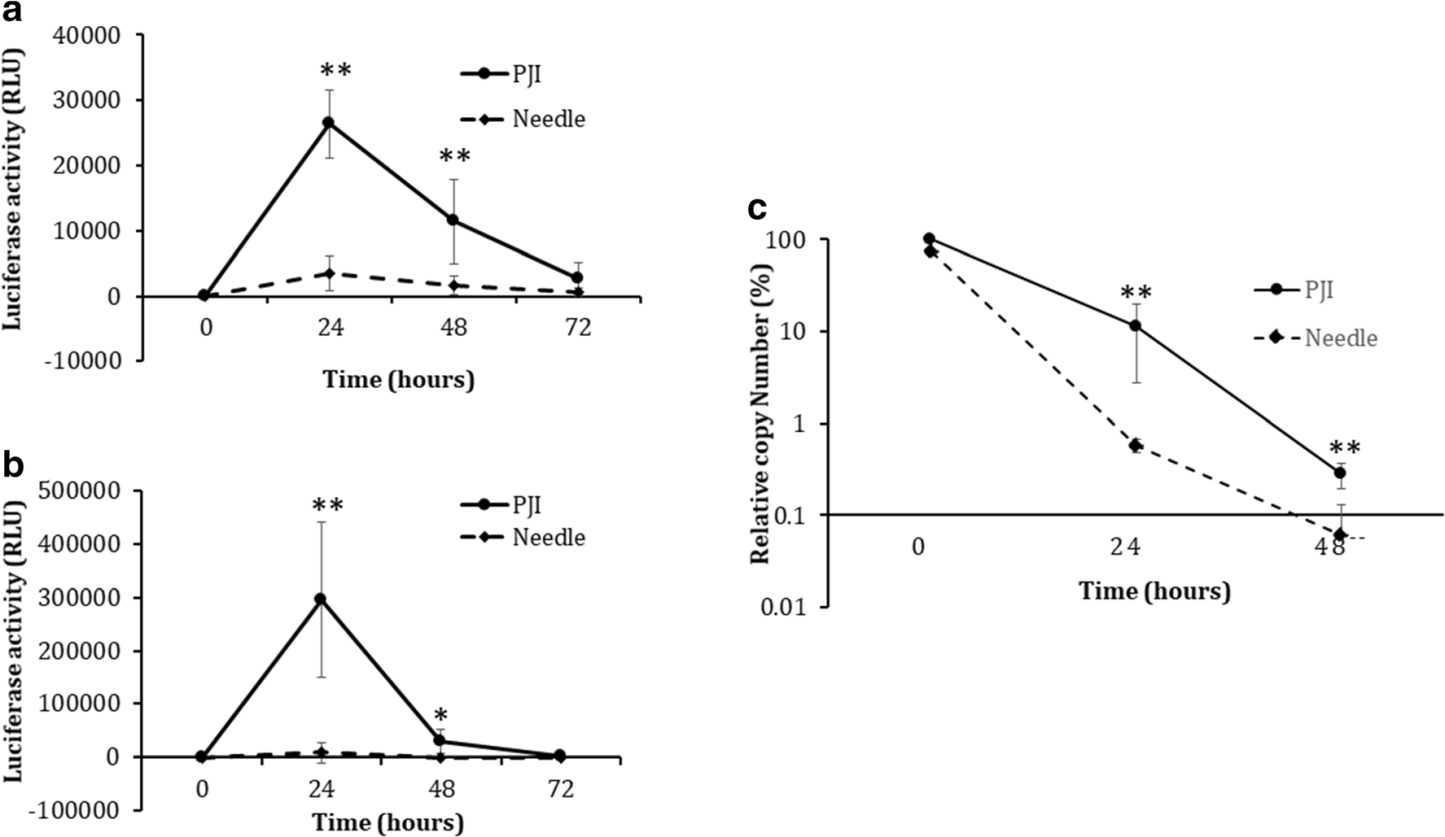
Luciferase expression by pyro-drive jet injector (PJI) or needle syringe injection and plasmid DNA copy number. Luc plasmid DNA was injected *via* PJI or needle syringe into mice (*n* = 6) (**a**) and rats (*n* = 4) (**b**). After injection, skin samples were collected every 24 h from 0 to 72 h, and a luciferase assay was performed. The Luc plasmid DNA was injected by the PJI or needle syringe into rats (*n* = 3) (**c**). After injection, skin samples were collected every 24 h from 0 to 48 h, and relative copy numbers were analyzed by q-PCR (Student’s *t* test—*p < 0.05; **p < 0.01). Solid black line: Luc plasmid DNA injected in the PJI group; dashed black line: Luc plasmid DNA injected in the needle syringe group. *Y*-axis indicates the relative light unit (RLU) for (**a**) and (**b**), and relative copy number for (**c**) (mean ± standard deviation (SD))

Results from the Cy3-p induction study showed that PJI resulted in more efficient plasmid DNA distribution to the nucleus compared to that with the needle syringe. This experiment also evaluated potential differences in gene expression between PJI and needle syringe delivery groups. Luc plasmid DNA injection in both mice (Fig. 4a) and rats (Fig. 4b) induced luciferase expression; however, the PJI group showed 7.4- and 36.3-fold higher luciferase expression levels in mice and rats, respectively, compared to those in the needle syringe delivery group. The PJI method was thus able to deliver plasmids resulting in successful expression of the target gene. Furthermore, an investigation of relative plasmid DNA copy numbers based on recovered DNA from Luc plasmid DNA injection sites differed significantly between the PJI and needle syringe methods at 24 h (*p* < 0.01) and 48 h (*p* < 0.01). Following PJI, the plasmid DNA copy numbers decreased from 100% to 11.27% at 24 h and 0.28% at 48 h, whereas in the syringe delivery group, they decreased from 75.39% to 0.58% at 24 h and 0.06% at 48 h (when plasmid DNA copy numbers at 0 h were set to 100% based on the PJI group; Fig. 4c). Based on the decrease in the ratio of plasmid DNA copy numbers, there was a statistically significant difference between PJI and needle syringe groups at 24 (*p* < 0.01) and 48 h (*p* < 0.01). Specifically, there was an approximate 15-fold difference between the two delivery methods, 100% to 11.27% by PJI and 75.39% to 0.58% by needle syringe, at 24 h. Furthermore, from 24 to 48 h, the plasmid DNA copy number decreased to less than one tenth in both cases. Results thus showed that the injected plasmid DNA copy number followed the same pattern as gene expression. In summary, luciferase expression was highest at 24 h, after which point it consistently decreased until 72 h; further, the expression patterns between rats and mice were similar. These results clearly indicate the potential of the PJI to be used as an intradermal DNA expression device.

### Model DNA Vaccination Experiment

**Fig. 5:**
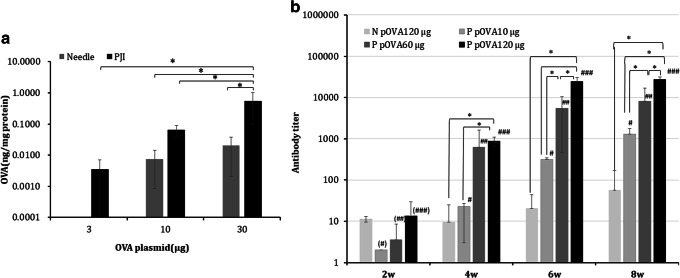
Ovalbumin (OVA) protein and anti-OVA antibody production. pOVA was injected by either the pyro-drive jet injector (PJI) or a 27G needle syringe. OVA protein expression and anti-OVA antibody were then measured. **a**Twenty-four hours after 3, 10, or 30 μg of pOVA injection, the injected skin was excised, and OVA protein expression levels were measured (*n* = 3). *p < 0.05 (Dunnett’s test). **b** pOVA (10, 60, or 120 μg) was injected by PJI and 120 μg pOVA was injected by a 27G needle syringe three times over a 2-week period. The anti-OVA serum antibody was evaluated every 2 weeks until 8 weeks post-injection. N pOVA 120 μg: 120 μg pOVA was injected by needle syringe (*n* = 4); P pOVA 10 μg: 10 μg pOVA was injected by PJI (*n* = 4); P pOVA 60 μg: 60 μg pOVA was injected by PJI (*n* = 4); P pOVA 120 μg: 120 μg pOVA was injected by PJI (*n* = 4). # indicates a statistically significant difference (*p* < 0.05) in antibody titers between week 2 and weeks 4, 6, and 8 of the 10 μg pOVA injected group, ## indicates a statistically significant difference (*p* < 0.05) in antibody titers between week 2 and weeks 4, 6, and 8 of the 60 μg pOVA injected group. ### indicates a statistically significant difference (*p* < 0.05) in antibody titers between week 2 and weeks 4, 6, and 8 of the 120 μg pOVA injected group; *p < 0.05 (Shirley–Williams test); *X*-axis indicates the experimental week and *Y*-axis indicates the antibody titer (mean ± SD)

To evaluate the potential of the PJI system for use as an intradermal DNA vaccination device, we selected pOVA as a model antigen and evaluated initial gene expression in the intradermal region. In rats, when the PJI was used to inject pOVA, a dose-dependent increase in OVA protein expression, specifically 0.56 ng/mg protein for 30 μg pOVA, 0.06 ng/mg protein for 10 μg pOVA, and 0.004 ng/mg protein for 3 μg pOVA, was observed, whereas low-level expression (0.02 ng/mg protein for 30 μg pOVA) was observed when the needle syringe was employed (Fig. 5a). Therefore, the PJI method effectively delivered plasmids and induced protein expression. Next, we sought to evaluate whether the PJI could be used for DNA vaccination. Thus, we compared antibody titer levels between the PJI and needle syringe injection methods. An antibody titer below 100 was observed after three injections using the needle syringe (Fig. 5b). However, in the PJI-injected groups, anti-OVA antibodies were detected in a dose-dependent manner at every pOVA injection dose (10, 60, and 120 μg), with a maximum antibody titer of 27,564. Furthermore, antibody production at 8 weeks was higher than that at 2 weeks for all tested pOVA injection doses (Fig. 5b). Similarly, in the mouse model, dose-dependent antibody production was observed (Supplementary Fig. 4). These results indicate that the PJI induces a DNA-encoded protein-specific antibody response in a dose-dependent manner, whereas DNA injection using a needle syringe did not efficiently induce specific antibodies.

In addition, the antibody titer of the 10 μg pOVA PJI-injected group was comparable to that of the 60 μg OVA recombinant protein needle syringe-injected group, while the 120 μg pOVA PJI-injected group exhibited a significantly higher antibody titer than that of the 60 μg OVA recombinant protein needle syringe-injected group at 8 weeks (Supplementary Fig. 3).

Following use of 35 mg ignition powder (the optimal condition for intradermal injection), a more stable antibody titer was achieved compared to that with 90 mg injection powder, resulting in distribution of India ink from the intradermal to musculi trunci region (Fig. 1). Further, 35 mg of injection powder resulted in a similar order of antibody titers, specifically, 10,206, 25,715, and 31,973 for each rat, whereas the values became much more variable following use of 90 mg injection powder (13, 1095, and 33,011). These results suggest that injection depth may affect antibody production. Specifically, accurate intradermal injection results in effective stable antibody production (Supplementary Fig. 5).

## DISCUSSION

Recently developed DNA vaccines have been proposed as a promising new vaccination method [18–20]. In traditional vaccine production, antigen preparation has required the cultivation of pathogenic bacteria or viruses to obtain the appropriate bacterial or viral strains. Compared to traditional vaccines, the DNA vaccine manufacturing process is simple. With the progress made in DNA analysis techniques, DNA vaccines are easier to design and produce; however, the DNA vaccine injection device requires further optimization. Herein, we not only highlight the novel adjustable PJI but also demonstrate the ability of this device to effectively deliver DNA vaccines. Animal models are required to test the effectiveness and ensure safety of newly developed medical therapeutic and prevention strategies.

Traditionally, many vaccines, including that for influenza, have been administrated intramuscularly *via* a needle syringe, despite the fact that APCs, such as LH cells, are more commonly found in the epidermal and dermal regions [21, 22]. Thus, a study comparing the vaccination efficacy of intramuscular and intradermal administration was conducted and reported [23]. Additionally, an intradermal vaccine as well as novel delivery techniques, including micro-needle devices, have been developed and investigated to improve vaccination therapy effectiveness [24–31]. One of the obstacles facing intradermal vaccine development is the technical difficulty associated with its delivery in animal models; as the skin thicknesses of rats and mice are approximately 2 and 0.8 mm, respectively, they are ideal candidates for initial experimentation [32]. Micro-needle devices have the potential to introduce vaccines into the intradermal region, however, cannot be commonly used for DNA vaccination [33]. Thus, an efficient intradermal plasmid DNA delivery and expression system is needed for new DNA vaccine methods. Sites of high APC distribution have always been considered as optimal injection targets; the intra-dermal zone contains mass distribution of APCs and is, therefore, a suitable target to induce cell-mediated immunity. The intra-dermal zone is also a good injection target; however, injection into this site requires a high level of technical proficiency. Jet injection has a low technical threshold yet the injection powers in existing products are static, with only a few exceptions. Nikola et al. reported depth-controllable computer-regulated injection and Miyazaki et al. proposed a PJI device with an adaptable injection depth [15, 16].

In the current study, we tested a new type of needleless jet injector and demonstrated its application potential in two animal models. As shown in our results (Fig. 1), we demonstrate the relationship between injection depth and injector power, which is adjustable based on the specific needs of the user by changing the ignition powder amount. When 15 mg of ignition powder was used, a larger amount of un-injected liquid was observed on the skin compared to that with 35 mg. Furthermore, in preliminary experiments, we observed that PJI did not break the skin surface in specific cases when 15 mg of ignition power was employed for rats (data not shown). However, with 35 mg, this issue was resolved. In contrast, when 55 mg of ignition powder was employed, the injected liquid diffused from the dermal region to the skin muscle region (Fig. 1a).

When Cy3-p was injected into the intradermal region of rats or mice *via* PJI, the plasmid DNA appeared to spread evenly into the dermal region, and 3D fluorescence microscopic analysis indicated that the PJI effectively introduced plasmid DNA into the nucleus immediately after injection. Interestingly, the injected plasmid DNA spread in concentric circles over a 3-mm diameter, despite the injection nozzle diameter being less than 0.2 mm. The reason for this diffusion pattern is unclear; however, a previous ultra-high-speed video analysis of a polyurethane gel experiment suggested that PJI-injected liquids spread both vertically and horizontally from the injection point [16]. Thus, the high-speed liquid flow generated by the ignition powder may have allowed for this even distribution throughout the dermal area. Alternatively, when Cy3-p was injected into the intradermal region of rats using a needle syringe, the plasmid DNA appeared to spread primarily within the central area of the dermis, whereas Cy3-p spread from the epidermis to the dermis following injection *via* PJI.

In the evaluation of skin damage, no significant differences were observed between the PJI and needle syringe groups at 24 h. However, small spherical cleavages were visible only in the PJI group until 6 h after injection. The same structural change was observed with another type of compressed gas-powered jet injector [8]. Thus, these characteristic structural changes might be caused by a certain type of jet injector (Fig. 3). In this study, the PJI was proven to be safe and reliable in animal experiments; the PJI successfully delivered injections into the intradermal region, without inducing remarkable adverse events such as structural changes in tissue or severe inflammation after 24 h in rats and mice.

To examine the application of the PJI for DNA vaccine delivery, we injected Luc plasmid DNA and analyzed luciferase expression to confirm plasmid DNA delivery and expression. The PJI method resulted in 7.4- and 36.3-fold higher luciferase expression than that of the needle syringe injection for mice and rats, respectively. The cause of this disparity in protein expression between PJI and needle syringe injection is currently unclear; however, we postulate that differences in flow rate between the two methods may be contributing to this observation. The injection power of PJI is primed by gunpowder, hence injection occurs at very rapid flow rate of approximately 1 mL/s [16], whereas the flow rate for normal injection *via* a needle syringe is less than 0.025 mL/s [34]. This report stated that the injection flow rate affects subsequent protein expression. Thus, PJI high injection speed may account for the observed increase in protein expression compared to that with needle syringe.

The Cy3-p study demonstrated that plasmid DNA merged with the nucleus following injection with the PJI device, indicating that PJI effectively introduces plasmid DNA into the nucleus at a higher frequency than does a needle syringe. These results were confirmed by the gene expression efficiency associated with each device. When the Luc plasmid DNA was injected by PJI, maximum luciferase expression was obtained 24 h after injection, and activity rapidly decreased in a time-dependent manner (Fig. 4a, b). Plasmid DNA copy numbers also decreased in a time-dependent manner (Fig. 4c). Interestingly, the decreasing ratio of plasmid DNA copy numbers after needle syringe injection occurred faster than that with PJI. Although the reason for this difference is unclear, the differing efficiencies observed between these devices in introducing plasmid DNA into the nucleus may be contributing to this effect.

Figure 4a and b demonstrates that PJI injection can induce luciferase gene expression; however, the process of DNA vaccination is more complex than that associated with simple plasmid DNA expression. We, therefore, tested the ability of PJI to induce antibody production using pOVA as a DNA vaccination model in three experiments. We confirmed OVA expression and dose-dependent anti-OVA antibody production in the pOVA groups injected *via* PJI, whereas a low antibody titer (below 100) was observed when pOVA was injected using a needle syringe (Fig. 5a, b). In addition, we showed the 10 μg of pOVA induced a comparable antibody titer to that of 60 μg OVA protein (Supplementary Fig. 3). Furthermore, the results based on different ignition powder conditions, specifically, 35 mg for optimal intradermal injection and 90 mg for intradermal to musculi trunci injection, clearly demonstrated the importance of accurate DNA distribution for induction of stable antibody production. Both conditions induced antibody production; however, a large degree of variability (13 to 33,011) was observed in antibody titer when 90 mg of ignition powder was used. Alternatively, the antibody titer was in the same order of magnitude for all three replicates when 35 mg ignition powder was used (Supplementary Fig. 4). Furthermore, dose-dependent antibody production was observed in the mouse model (Supplementary Fig. 5). These results collectively suggest that all processes, from nuclear plasmid DNA induction, protein expression in the skin region, immune system stimulation, and dose-dependent antibody production, are effectively induced by injection with a PJI device.

## CONCLUSIONS

We evaluated the efficacy of the PJI system as an intradermal DNA vaccination device, and the results show that PJI effectively delivers plasmid DNA into the nuclei of the dermal region and induces efficient gene expression. Furthermore, a model DNA vaccination study showed dose-dependent and stable antibody production. Thus, PJI should be considered as a novel DNA vaccination device for larger animals; however, further testing must be performed prior to large animal application. Overall, this study clearly demonstrates that the PJI system is a promising new DNA vaccine delivery device.

## Electronic supplementary material

Supplementary Fig. 1 Recommend Injection Region. The blue colored area indicates the suitable injection area; the yellow colored area is not a suitable injection area (PNG 594 kb)

High resolution image (TIF 116 kb)

Supplementary Fig. 2 Time course of skin damage test. Luciferase plasmid DNA was injected using 25 mg of ignition powder by the pyro-drive jet injector (PJI; Device) (**a**) or a 30G needle syringe (**b**). After injection, the skin samples were collected at 0 h (just after injection), 6 h, and 24 h and stained using H&E. Blue arrows: visible puncture point. Black arrows: spherical cleavages (PNG 3304 kb)

High resolution image (TIF 539 kb)

Supplementary Fig. 3 Comparison of antibody induction between pOVA and OVA. pOVA (10 and 120 μg) was injected by the pyro-drive jet injector (PJI) and 60 μg of OVA recombinant protein was injected by a 27G needle syringe every 2 weeks for a total of three injections. The anti-OVA antibody in serum was collected and evaluated until 8 weeks. P pOVA 10 μg (*n* = 5): 10 μg pOVA was injected by the PJI (*n* = 5); P pOVA 120 μg: 120 μg pOVA was injected by the PJI (*n* = 5); N OVA 60 μg: 60 μg OVA recombinant protein was injected by needle syringe (*n* = 4); *p < 0.05 (Shirley–Williams test). *Y*-axis indicates the antibody titer (mean ± SD) (PNG 330 kb)

High resolution image (TIF 99 kb)

Supplementary Fig. 4 Anti-OVA antibody production. pOVA (1, 3.3, and 10 μg) was injected by the pyro-drive jet injector (PJI) and 10 μg of pOVA was injected by a 30G needle syringe every 2 weeks for a total of three injections. The anti-OVA antibody in serum was collected and evaluated until 8 weeks. P pOVA 10 μg: 10 μg pOVA was injected by the PJI; P pOVA 3.3 μg: 3.3 μg pOVA was injected by the PJI; P pOVA 1 μg: 1 μg pOVA was injected by the PJI; N OVA 10 μg: 10 μg pOVA was injected by a needle syringe; *p < 0.05 (Shirley–Williams test). *Y*-axis indicates the antibody titer (mean ± SD) (PNG 419 kb)

High resolution image (TIF 124 kb)

Supplementary Fig. 5 Relationship between ignition powder amount and antibody production. Sixty micrograms of pOVA was injected over a 2-week period for a total of three injections using two different ignition powder conditions (35 and 90 mg). Serum was collected every 2 weeks for 6 weeks, and the serum anti-OVA antibody was evaluated (*n* = 3). ●: anti-OVA antibody titer for individual animal. *Y*-axis indicates the antibody titer (PNG 298 kb)

High resolution image (TIF 75 kb)

## References

[1] Weniger BG, Mark JP. Alternative vaccine delivery methods. In: Plotkin S, Orenstein W, Offit P, editors. Vaccines 6th. ELSEVIER; 2013. p. 1200–1231.

[2] Edens C, Dybdahl-Sissoko NC, Weldon WC, Oberste MS, Prausnitz MR (2015). Inactivated polio vaccination using a microneedle patch is immunogenic in the rhesus macaque. Vaccine..

[3] Gorres JP, Lager KM, Kong W-P, Royals M, Todd J-P, Vincent AL (2011). DNA vaccination elicits protective immune responses against pandemic and classic swine influenza viruses in pigs. Clin Vaccine Immunol.

[4] Williams J, Fox-Leyva L, Christensen C, Fisher D, Schlicting E, Snowball M, Negus S, Mayers J, Koller R, Stout R (2000). Hepatitis a vaccine administration: comparison between jet-injector and needle injection. Vaccine..

[5] Kutzler MA, Weiner DB (2008). DNA vaccines: ready for prime time?. Nat Rev Genet.

[6] Prather KJ, Sagar S, Murphy J, Chartrain M (2003). Industrial scale production of plasmid DNA for vaccine and gene therapy: plasmid design, production, and purification. Enzym Microb Technol.

[7] Lamolinara A, Stramucci L, Hysi A, Iezzi M, Marchini C, Mariotti M, et al. Intradermal DNA electroporation induces cellular and humoral immune response and confers protection against HER2/neu tumor. J Immunol Res. 2015. 10.1155/2015/159145. 10.1155/2015/159145 PMC4515534 26247038

[8] Kwon TR, Seok J, Jang JH, Kwon MK, Oh CT, Choi EJ, Hong HK, Choi YS, Bae J, Kim BJ (2016). Needle-free jet injection of hyaluronic acid improves skin remodeling in a mouse model. Eur J Pharm Biopharm.

[9] Cattamanchi A, Posavad CM, Wald A, Baine Y, Moses J, Higgins TJ, Ginsberg R, Ciccarelli R, Corey L, Koelle DM (2008). Needle-free injection system to healthy, HSV-2-seronegative adults by a type 2 (HSV-2) DNA vaccine administered phase I study of a herpes simplex virus. Clin Vaccine Immunol.

[10] Beckett CG, Tjaden J, Burgess T, Danko JR, Tamminga C, Simmons M (2011). Evaluation of a prototype dengue-1 DNA vaccine in a phase 1 clinical trial. Vaccine.

[11] Ault A, Zajac AM, Kong WP, Gorres JP, Royals M, Wei CJ, Bao S, Yang ZY, Reedy SE, Sturgill TL, Page AE, Donofrio-Newman J, Adams AA, Balasuriya UB, Horohov DW, Chambers TM, Nabel GJ, Rao SS (2012). Immunogenicity and clinical protection against equine influenza by DNA vaccination of ponies. Vaccine..

[12] Bråve A, Gudmundsdotter L, Sandström E, Haller BK, Hallengärd D, Maltais AK, King AD, Stout RR, Blomberg P, Höglund U, Hejdeman B, Biberfeld G, Wahren B (2010). Biodistribution, persistence and lack of integration of a multigene HIV vaccine delivered by needle-free intradermal injection and electroporation. Vaccine..

[13] Hallengärd D, Bråve A, Isaguliants M, Blomberg P, Enger J, Stout R (2012). A combination of intradermal jet-injection and electroporation overcomes in vivo dose restriction of DNA vaccines. Genet Vaccines Ther.

[14] Borggren M, Nielsen J, Bragstad K, Karlsson I, Krog JS, Williams JA, Fomsgaard A (2015). Vector optimization and needle-free intradermal application of a broadly protective polyvalent influenza a DNA vaccine for pigs and humans. Hum Vaccin Immunother.

[15] Kojic N, Goyal P, Lou CH, Corwin MJ (2017). An innovative needle-free injection system: comparison to 1 ml standard subcutaneous injection. AAPS PharmSciTech.

[16] Miyazaki H, Atobe S, Suzuki T, Iga H, Terai K (2019). Development of pyro-drive jet injector with controllable jet pressure. J Pharm Sci.

[17] Diebold SS, Cotten M, Koch N, Zenke M (2001). MHC class II presentation of endogenously expressed antigens by transfected dendritic cells. Gene Ther.

[18] Andersen TK, Zhou F, Cox R, Bogen B, Grødeland G (2017). A DNA vaccine that targets hemagglutinin to antigen-presenting cells protects mice against H7 influenza. J Virol.

[19] Lee H, Jeong M, Oh J, Cho Y, Shen X, Stone J, Yan J, Rothkopf Z, Khan AS, Cho BM, Park YK, Weiner DB, Son WC, Maslow JN (2017). Preclinical evaluation of multi antigenic HCV DNA vaccine for the prevention of hepatitis C virus infection. Sci Rep.

[20] Yang FQ, Rao GR, Wang GQ, Li YQ, Xie Y, Zhang Z-Q, Deng CL, Mao Q, Li J, Zhao W, Wang MR, Han T, Chen SJ, Pan C, Tan DM, Shang J, Zhang MX, Zhang YX, Yang JM, Chen GM (2017). Phase IIb trial of in vivo electroporation mediated dual-plasmid hepatitis B virus DNA vaccine in chronic hepatitis B patients under lamivudine therapy. World J Gastroenterol.

[21] Seneschal J, Clark RA, Gehad A, Baecher-Allan CM, Kupper TS (2012). Human epidermal Langerhans cells maintain immune homeostasis in skin by activating skin resident regulatory T cells. Immunity..

[22] Abd Warif NM, Stoitzner P, Leggatt GR, Mattarollo SR, Frazer IH, Hibma MH (2015). Langerhans cell homeostasis and activation is altered in hyperplastic human papillomavirus type 16 E7 expressing epidermis. PLoS One.

[23] Ivan FN, Hung K-YY (2018). Immunogenicity, safety and tolerability of intradermal influenza vaccines. Hum Vaccin Immunother.

[24] Meijer WJ, Westing AMJ, Bos AA, Kuiphuis JCF, Hagelen EMM, Wilschut JC, de Vries MJT, Riezebos-Brilman A (2017). Influenza vaccination in healthcare workers; comparison of side effects and preferred route of administration of intradermal versus intramuscular adoministration. Vaccine.

[25] Denis M, Knezevic I, Wilde H, Hemachudha T, Briggs D, Knopf L. An overview of the immunogenicity and effectiveness of current human rabies vaccines administered by intradermal route. Vaccine. 2018. 10.1016/j.vaccine.2018.11.072. 10.1016/j.vaccine.2018.11.072 30551985

[26] Göller M, Fels M, Gerdts W-R, Kemper N (2018). Intradermal versus intramuscular vaccine application in suckling piglets—comparison with regard to dermal reactions, performance and procedural aspects. Tierarztl Prax Ausg G.

[27] Kim NY, Ahn HB, Yu CH, Song DH, Hur GH, Shin YK (2018). Intradermal immunization with botulinum neurotoxin serotype E DNA vaccine induces humoral and cellular immunity and protects against lethal toxin challenge. Hum Vaccin Immunother.

[28] Kim YC, Park J, Prausnitz MR (2012). Microneedles for drug and vaccine delivery. Adv Drug Deliv Rev.

[29] Kim D-H, Jang EH, Lee KJ, Lee JY, Park SH, Seo IH (2017). A biodegradable microneedle cuff for comparison of drug effects through perivascular delivery to balloon-injured arteries. Polymers..

[30] Niu L, Chu LY, Burton SA, Hansen KJ, Panyam J (2019). Intradermal delivery of vaccine nanoparticles using hollow microneedle array generates enhanced and balanced immune response. J Control Release.

[31] Gala RP, Zaman RU, D’Souza MJ, Zughaier SM (2018). Novel whole-cell inactivated Neisseria gonorrhoeae microparticles as vaccine formulation in microneedle-based transdermal immunization. Vaccines..

[32] Todo H (2017). Transdermal permeation of drugs in various animal species. Pharmaceutics.

[33] Shende P, Sardesai M, Gaud RS (2018). Micro to nanoneedles: a trend of modernized transepidermal drug delivery system. Artif Cells Nanomed Biotechnol.

[34] André FM, Cournil-Henrionnet C, Vernerey D, Opolon P, Mir LM (2006). Variability of naked DNA expression after direct local injection: the influence of the injection speed. Gene Ther.

