# Supplementary figures and images for: Stable Immune Response Induced by Intradermal DNA Vaccination by a Novel Needleless Pyro-Drive Jet Injector
Source: AAPS PharmSciTech. 2019 Dec 9;21(1):19. doi: 10.1208/s12249-019-1564-z (PMC6901418; doi:10.1208/s12249-019-1564-z)

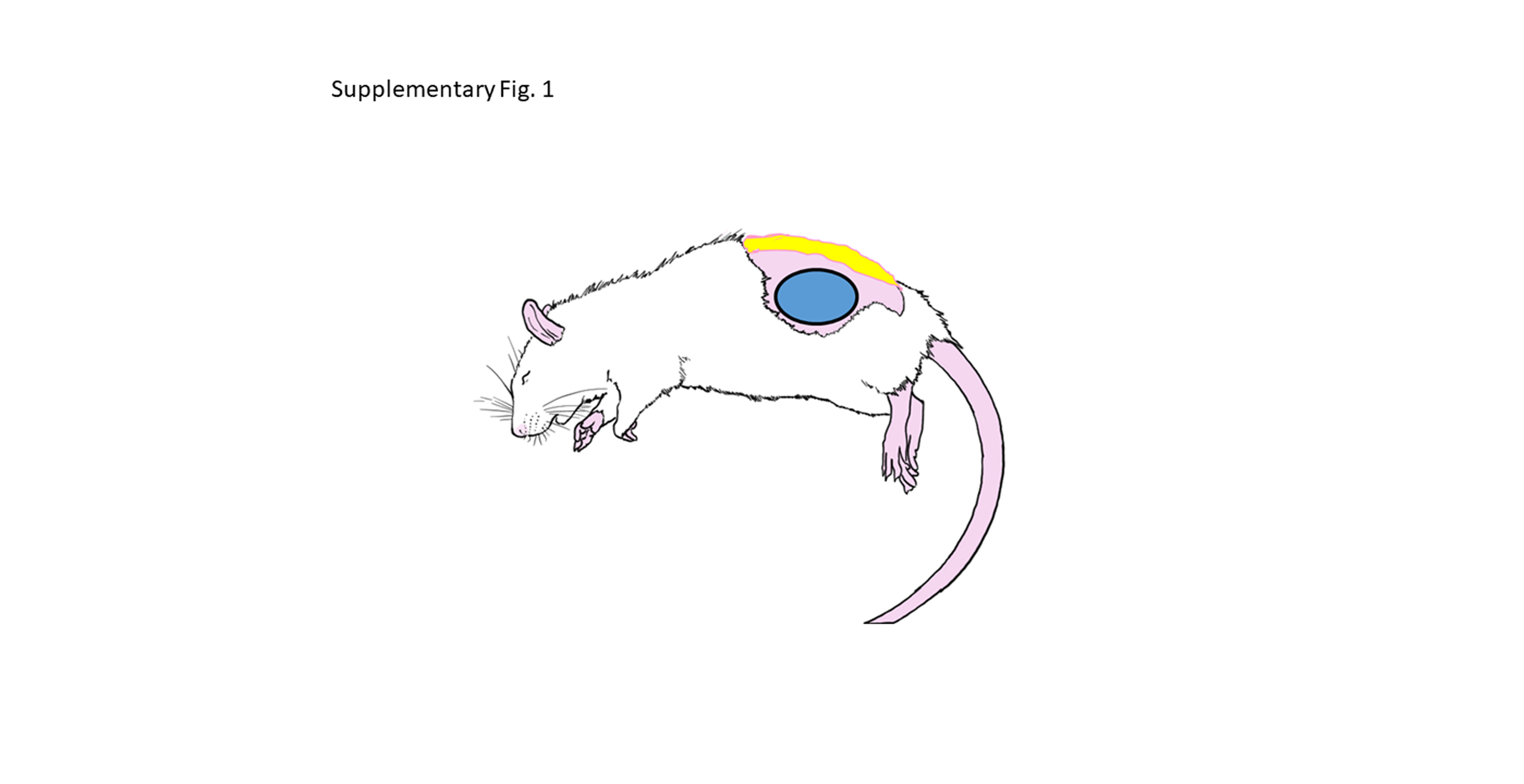

Supplement: Supplementary file 1 — Recommend Injection Region. The blue colored area indicates the suitable injection area; the yellow colored area is not a suitable injection area (PNG 594 kb) [file 12249_2019_1564_Fig6_ESM.png]

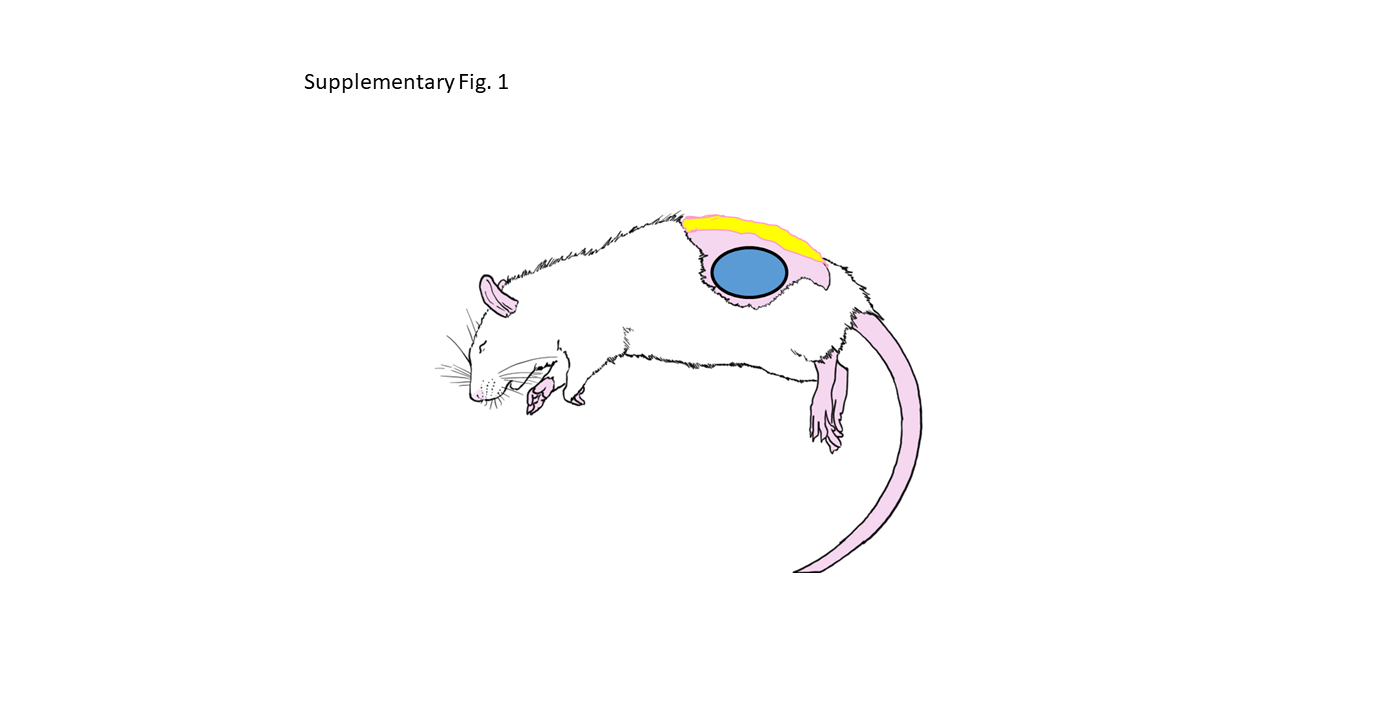

Supplement: Supplementary file 2 — High resolution image (TIF 116 kb) [file 12249_2019_1564_MOESM1_ESM.tif]

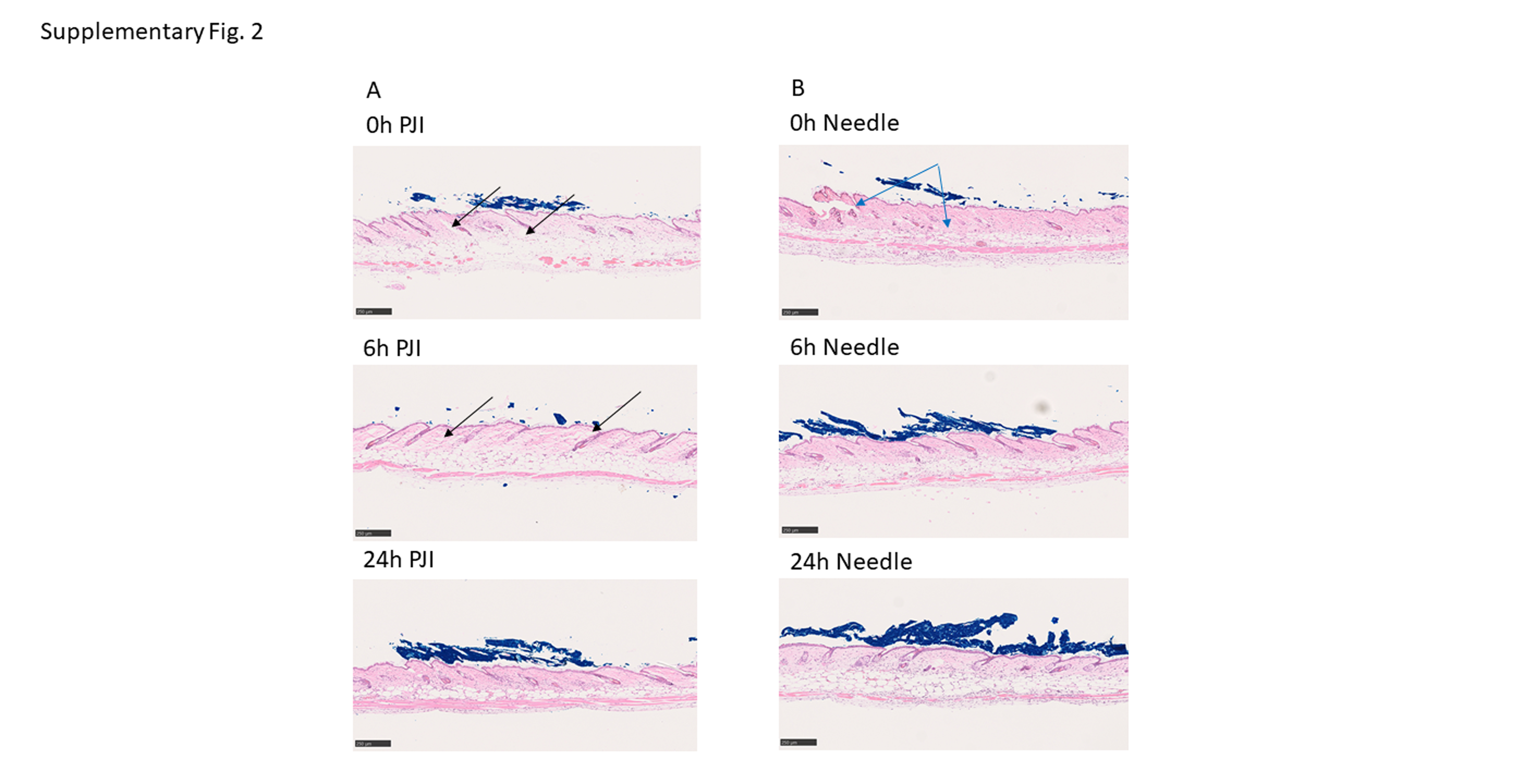

Supplement: Supplementary file 3 — Time course of skin damage test. Luciferase plasmid DNA was injected using 25 mg of ignition powder by the pyro-drive jet injector (PJI; Device) (a) or a 30G needle syringe (b). After injection, the skin samples were collected at 0 h (just after injection), 6 h, and 24 h and stained using H&E. Blue arrows: visible puncture point. Black arrows: spherical cleavages (PNG 3304 kb) [file 12249_2019_1564_Fig7_ESM.png]

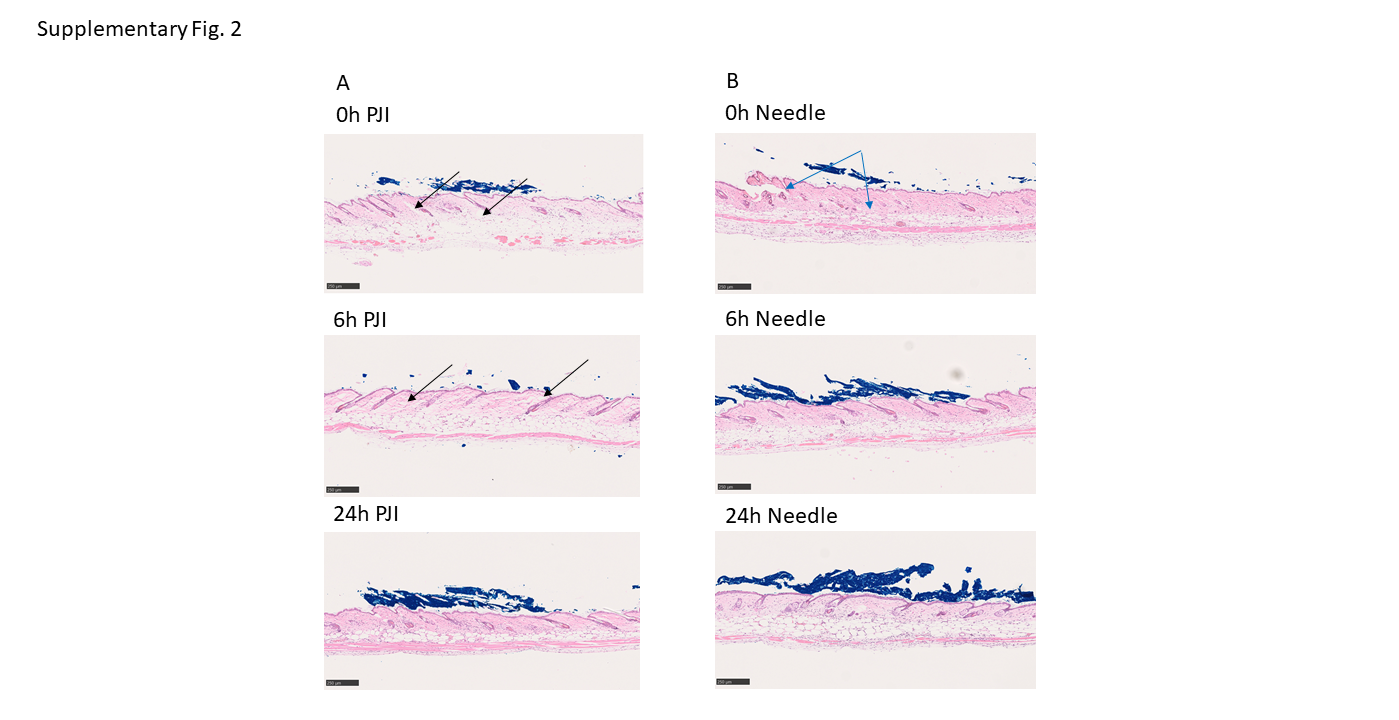

Supplement: Supplementary file 4 — High resolution image (TIF 539 kb) [file 12249_2019_1564_MOESM2_ESM.tif]

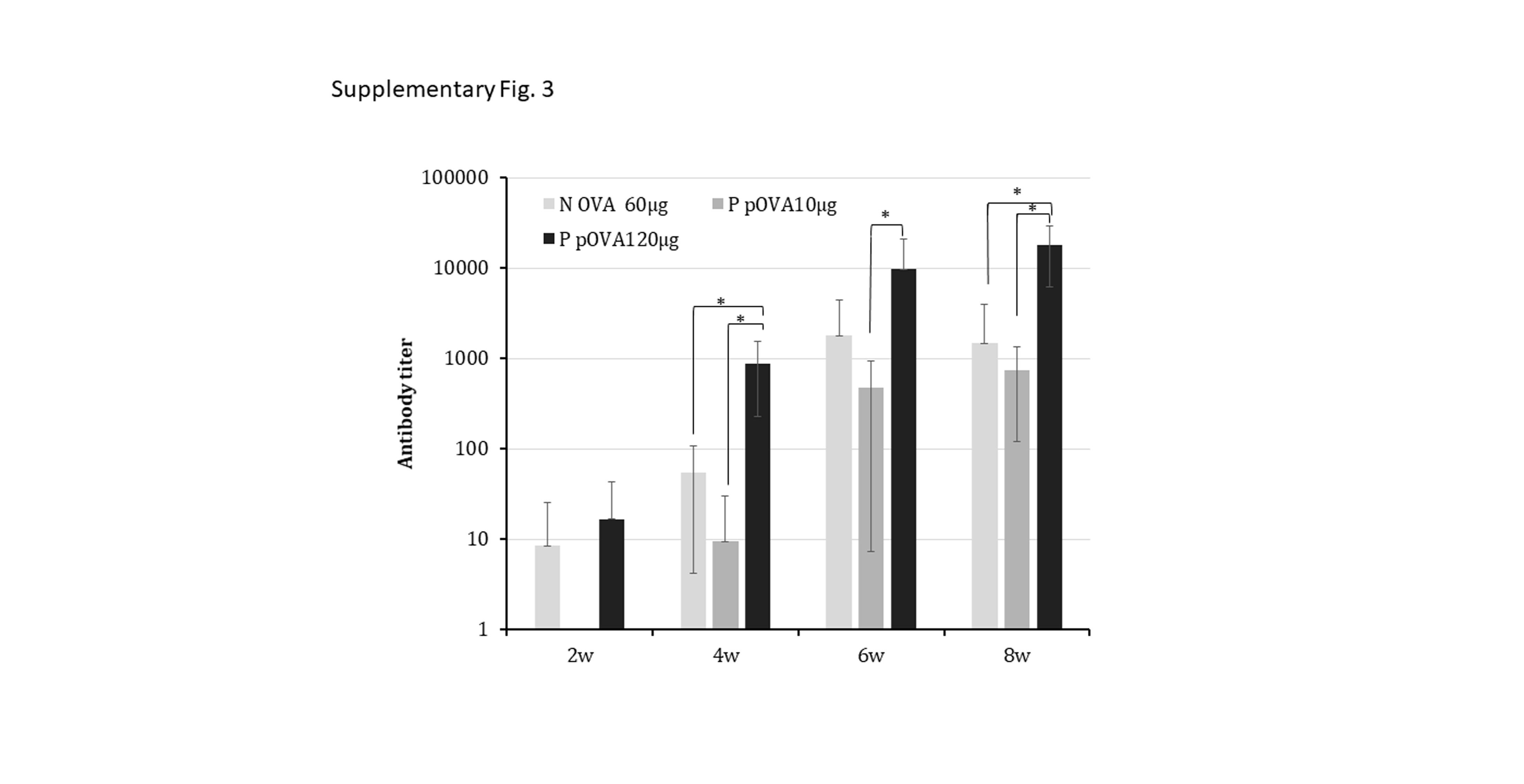

Supplement: Supplementary file 5 — Comparison of antibody induction between pOVA and OVA. pOVA (10 and 120 μg) was injected by the pyro-drive jet injector (PJI) and 60 μg of OVA recombinant protein was injected by a 27G needle syringe every 2 weeks for a total of three injections. The anti-OVA antibody in serum was collected and evaluated until 8 weeks. P pOVA 10 μg (n = 5): 10 μg pOVA was injected by the PJI (n = 5); P pOVA 120 μg: 120 μg pOVA was injected by the PJI (n = 5); N OVA 60 μg: 60 μg OVA recombinant protein was injected by needle syringe (n = 4); *p < 0.05 (Shirley–Williams test). Y-axis indicates the antibody titer (mean ± SD) (PNG 330 kb) [file 12249_2019_1564_Fig8_ESM.png]

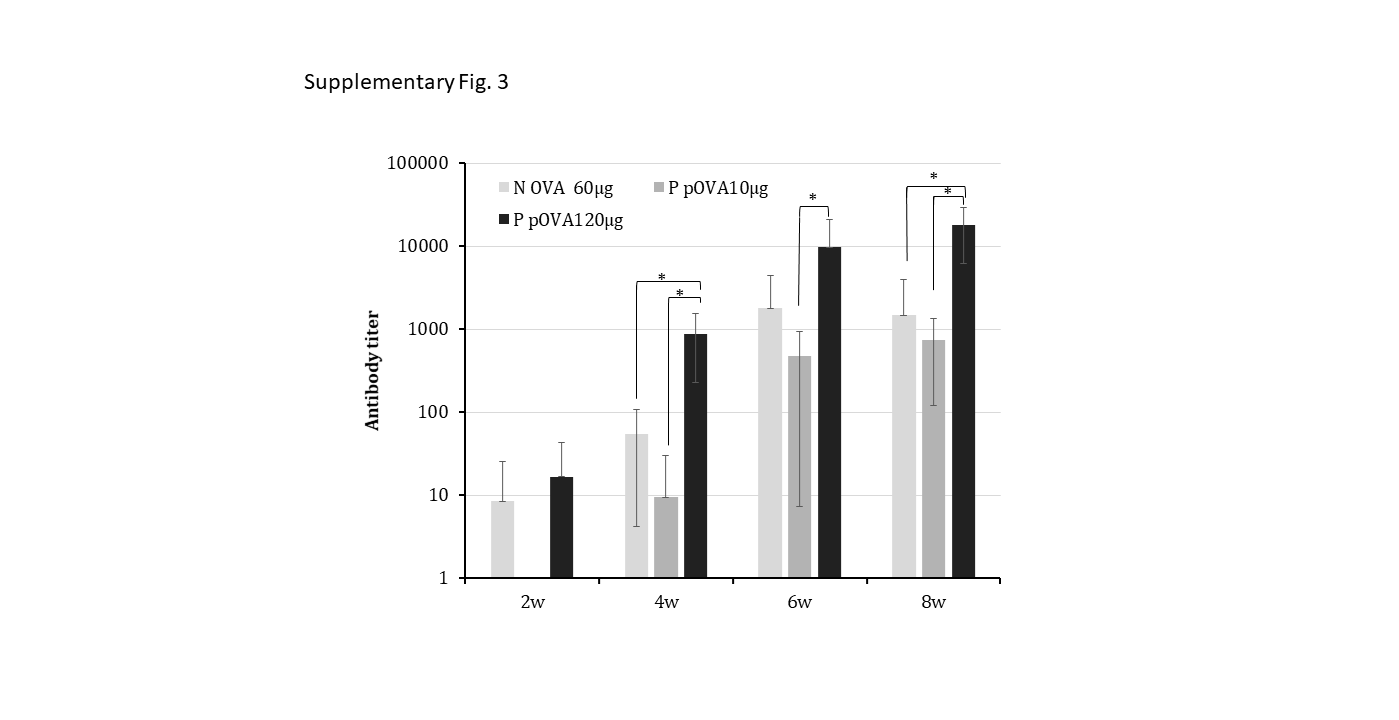

Supplement: Supplementary file 6 — High resolution image (TIF 99 kb) [file 12249_2019_1564_MOESM3_ESM.tif]

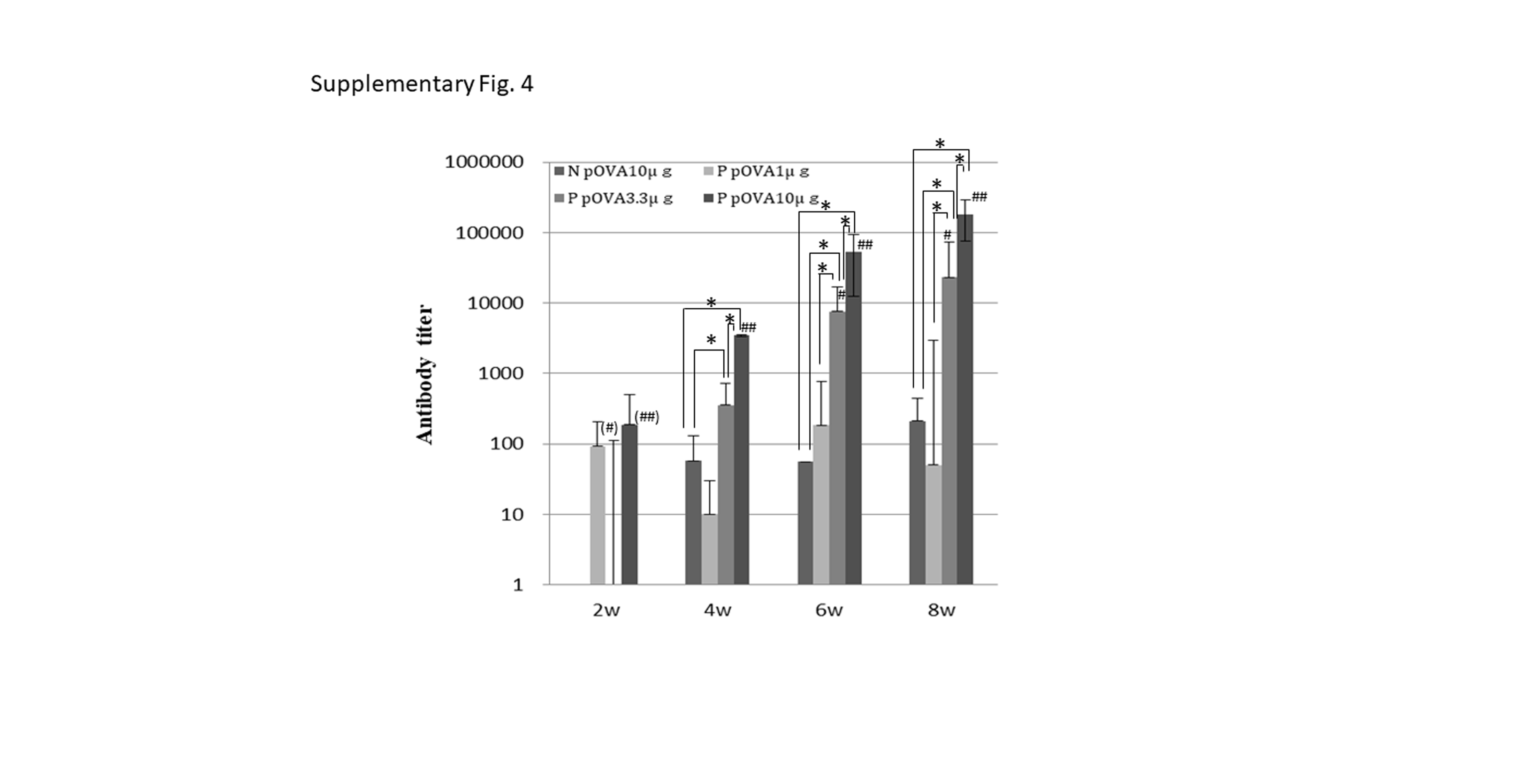

Supplement: Supplementary file 7 — Anti-OVA antibody production. pOVA (1, 3.3, and 10 μg) was injected by the pyro-drive jet injector (PJI) and 10 μg of pOVA was injected by a 30G needle syringe every 2 weeks for a total of three injections. The anti-OVA antibody in serum was collected and evaluated until 8 weeks. P pOVA 10 μg: 10 μg pOVA was injected by the PJI; P pOVA 3.3 μg: 3.3 μg pOVA was injected by the PJI; P pOVA 1 μg: 1 μg pOVA was injected by the PJI; N OVA 10 μg: 10 μg pOVA was injected by a needle syringe; *p < 0.05 (Shirley–Williams test). Y-axis indicates the antibody titer (mean ± SD) (PNG 419 kb) [file 12249_2019_1564_Fig9_ESM.png]

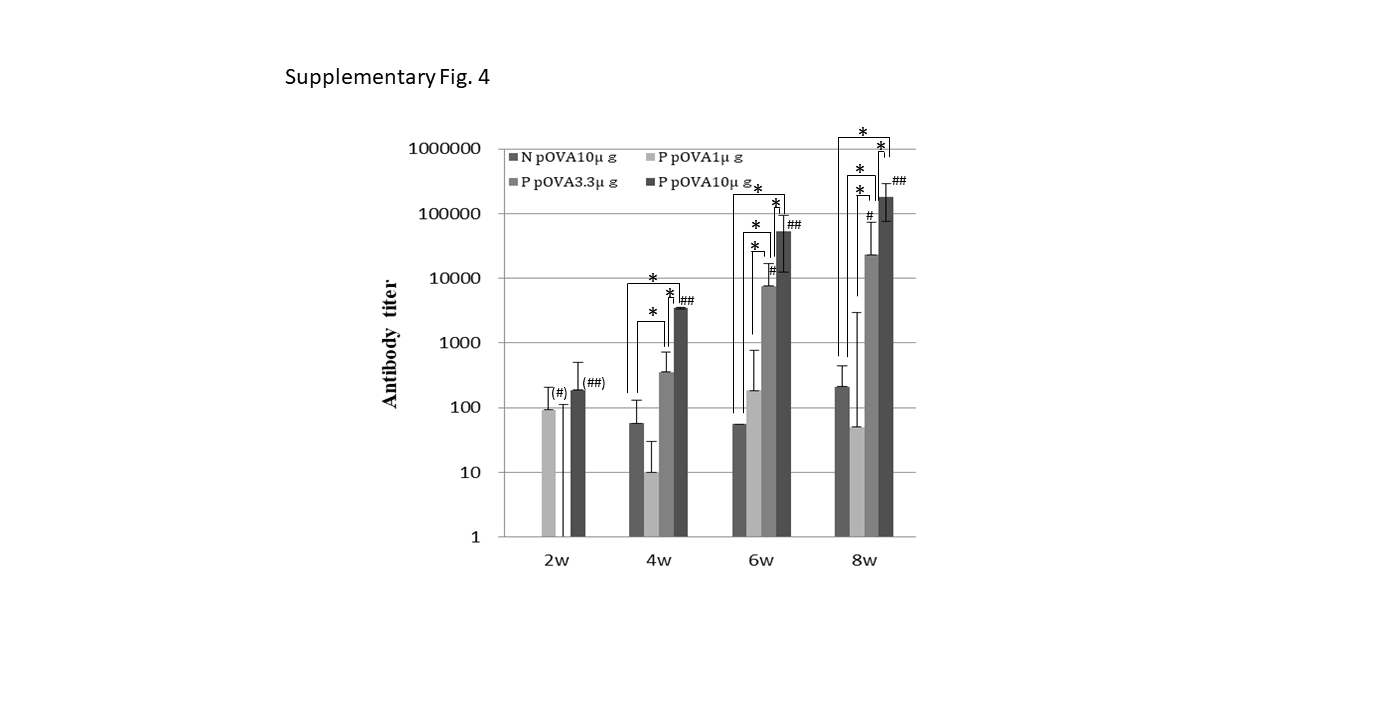

Supplement: Supplementary file 8 — High resolution image (TIF 124 kb) [file 12249_2019_1564_MOESM4_ESM.tif]

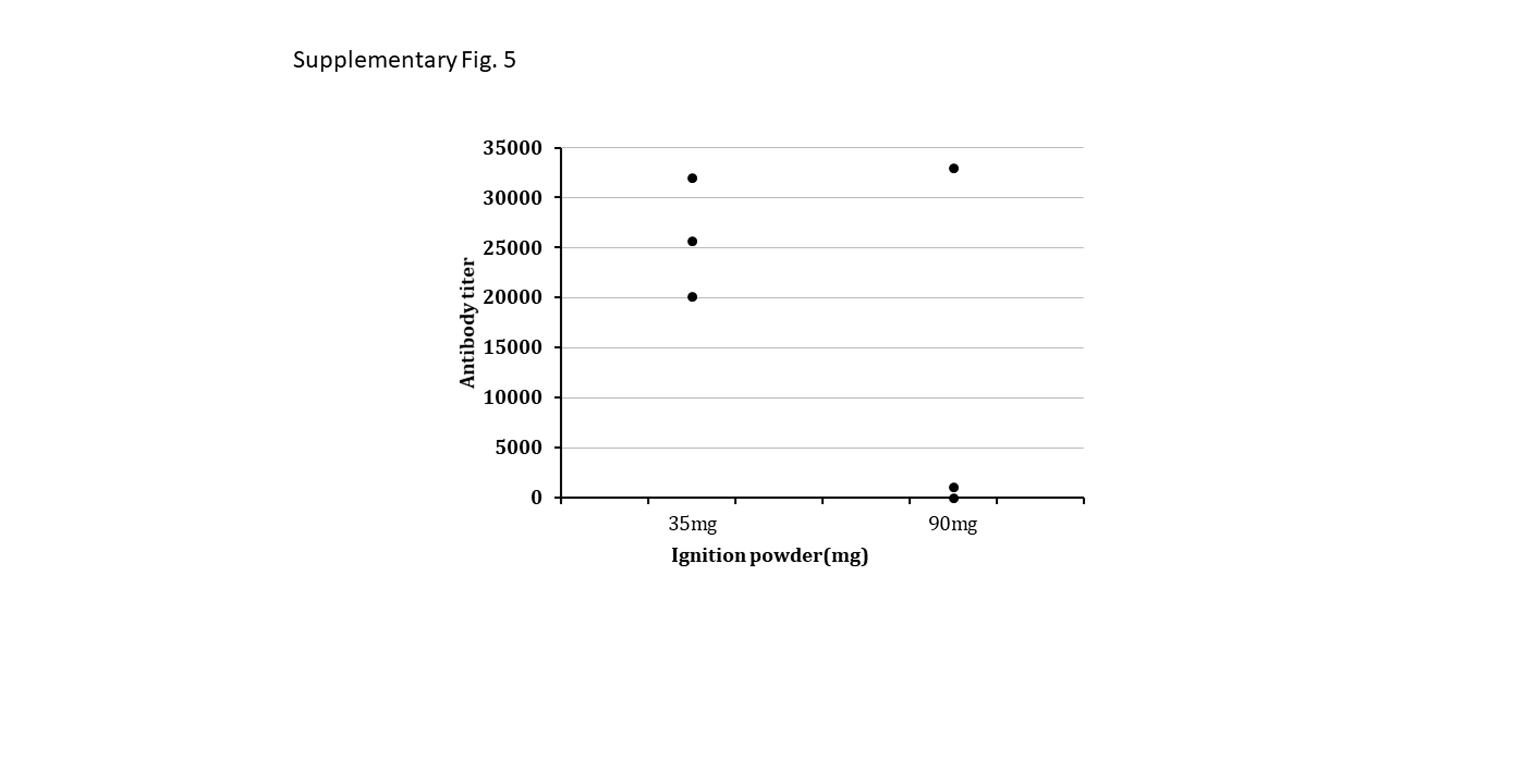

Supplement: Supplementary file 9 — Relationship between ignition powder amount and antibody production. Sixty micrograms of pOVA was injected over a 2-week period for a total of three injections using two different ignition powder conditions (35 and 90 mg). Serum was collected every 2 weeks for 6 weeks, and the serum anti-OVA antibody was evaluated (n = 3). ●: anti-OVA antibody titer for individual animal. Y-axis indicates the antibody titer (PNG 298 kb) [file 12249_2019_1564_Fig10_ESM.png]

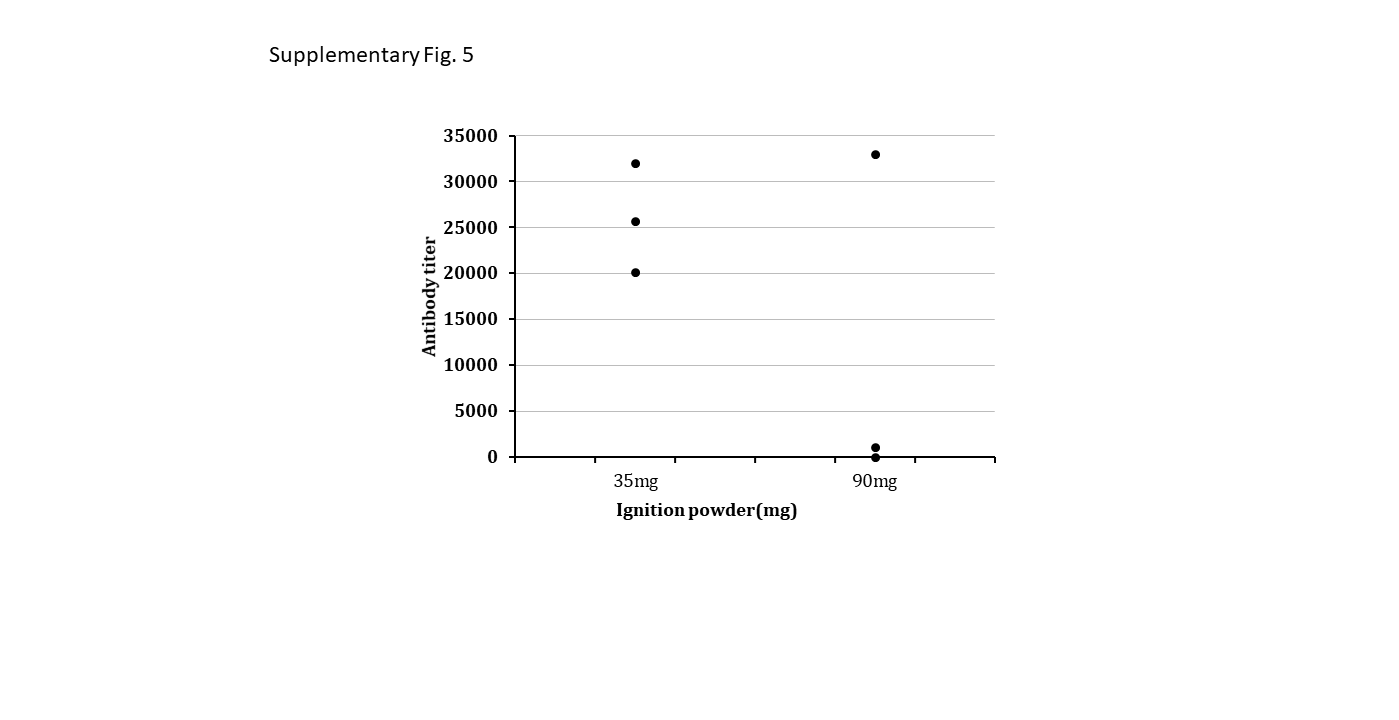

Supplement: Supplementary file 10 — High resolution image (TIF 75 kb) [file 12249_2019_1564_MOESM5_ESM.tif]
